# Commercialism in Medical Education in Low- and Middle-Income Countries through a Neo-colonial Lens: A Qualitative Evidence Meta-synthesis (2015-2025)

**DOI:** 10.12669/pjms.42.5.15253

**Published:** 2026-05

**Authors:** Abeera Fawad Khan, Ayesha Junaid, Junaid Sarfraz Khan

**Affiliations:** 1 Abeera Fawad Khan, School of Health Professionals’ Education, Research & Entrepreneurship, Health Services Academy, Islamabad, Pakistan; 2 Ayesha Junaid Centre for Language Development, Forman Christian College University, Lahore, Pakistan; 3 Junaid Sarfraz Khan, School of Health Professionals’ Education, Research & Entrepreneurship, Health Services Academy, Islamabad, Pakistan

**Keywords:** Accreditation, Commercialization, Continuing medical education, LMICs, Medical education, Neo-colonialism

## Abstract

Commercialization of medical education in low- and middle-income countries (LMICs) has intensified over the past decade, intersecting with neo-colonial power structures that shape training priorities, credentialing practices, and professional mobility. This review synthesizes qualitative evidence on how commercialism operates through neo-colonial mechanisms within LMIC medical education and assesses confidence in review findings using the GRADE-CERQual approach. A qualitative evidence meta-synthesis was conducted of peer-reviewed qualitative studies published between January 1, 2015 and August 15, 2025. The search was conducted on August 15, 2025, and included studies published online ahead of print where available.

Databases searched included PubMed/MEDLINE, Embase, Scopus, Web of Science, and ERIC. Eligible studies from Q1/Q2 journals were thematically synthesized in accordance with PRISMA guidance for qualitative reviews.

A total of four studies met strict inclusion criteria; therefore, findings should be interpreted as analytically illustrative and theory-generating rather than generalizable across all LMICs, which represent highly heterogeneous contexts. Four analytical themes were identified: credentialing dependence linked to migration markets, market-driven cross-border student recruitment, commercial sponsorship in continuing medical education, and accreditation functioning as a market signal. These mechanisms reinforce global inequities and may undermine socially accountable medical education. Strengthening regional accreditation systems, regulating industry involvement, and prioritizing social accountability are essential to rebalance medical education systems in LMICs.

***Abbreviations:*** **LMICs:** Low- and middle-income countries. **CME:** Continuing medical education. **CERQual:** Confidence in the Evidence from Reviews of Qualitative Research. **QES:** Qualitative evidence synthesis. **HICs:** High-income countries.

## INTRODUCTION

Over the past decade, medical education in low- and middle-income countries (LMICs) has been substantially reshaped by market-oriented forces. Private sector growth has accelerated, and students are paying to study. Pharmaceutical companies and corporations are investing significantly in continuing medical education (CME). These systems increasingly align with accreditation and credentialing models originating in high-income countries (HICs), reinforcing global standardization trends.

Professionalism is widely recognised as a core yet variably defined competency across health professions education, with substantial heterogeneity in how it is conceptualized and assessed.^1^ These dynamics often echo asymmetries from the past, rooted in colonial legacies. Scholars call this the “coloniality of power.” This refers to situations in which HIC knowledge, credentials, and labor markets are given more weight than LMIC institutions and learners. External benchmarks and commercial interests are used to control LMIC institutions and learners.

While LMICs are highly heterogeneous, this review aims to identify recurring mechanisms across selected contexts rather than claim universal applicability. Given the small number of eligible studies and the diversity of LMIC contexts, this review does not seek statistical or geographic generalization but instead identifies recurring mechanisms that may operate across settings in conceptually similar ways.

Foundational scholarship in global health and education has long argued that Western standards, epistemologies, and governance structures continue to shape professional training in the Global South.^2^,^3^ Building on this foundation, recent empirical studies have documented how marketization, credentialism, and accreditation regimes shape medical education systems in LMICs.^4^ Empirical qualitative studies illustrate these dynamics across multiple contexts. Research on English-medium medical programmes in China demonstrates how fee-paying international student recruitment, often mediated by commercial agents, reshapes curricula, quality assurance practices, and student risk exposure. Studies from Pakistan highlight tensions within continuing medical education (CME), where pharmaceutical sponsorship raises concerns regarding conflicts of interest and regulatory oversight. Critical discourse analyses further demonstrate how historical and contemporary credentialing narratives originating in high-income countries continue to shape opportunities, legitimacy, and professional trajectories for South Asian medical graduates.

Large entrance-exam coaching industries and recurring cheating scandals around high-stakes tests highlight how market incentives shape access to medical education and preparation. Although these reports do not constitute primary qualitative evidence, they provide important contextual insights into the commercial pressures affecting medical education systems.

### Rationale and Objectives:

***Rationale:*** Despite growing scholarly attention, there remains limited synthesized qualitative evidence examining how commercialism and neo-colonial power structures intersect to shape medical education in low- and middle-income countries. A qualitative evidence synthesis enables the identification of recurring mechanisms across diverse contexts, including credential dependency, sponsorship practices, and recruitment markets. Such synthesis can inform policy and regulatory approaches that are sensitive to both local educational needs and global structural inequalities.

### Objectives

1. Synthesize qualitative evidence (January 1, 2015 to August 15, 2025) from Q1/Q2 journals on how commercialism influences structures, practices, and experiences in LMIC medical education.
2. Interpret these influences through a neo-colonial lens to identify mechanisms and consequences.
3. Use GRADE-CERQual to rate confidence in each synthesized finding and outline implications for policy, regulation, and practice.

## METHODOLOGY

A systematic search was conducted for qualitative studies published between January 1, 2015 and August 15, 2025. Databases searched included PubMed/MEDLINE, Embase, Scopus, Web of Science, and ERIC. Search strategies combined terms related to medical education with terms relating to commercialism and marketization (e.g., commercial, private, for-profit, sponsorship, accreditation, recruitment) and qualitative methodologies.

### Ethical considerations:

This study is based exclusively on previously published literature and did not involve human participants or primary data collection. Therefore, formal ethical approval was not required. All included studies were peer-reviewed and conducted in accordance with established ethical standards.

### Eligibility criteria (SPIDER framework):

- ***Sample:*** Stakeholders in LMIC medical education (students, graduates, faculty, CME providers, regulators, agents).
- ***Phenomenon of Interest:*** Commercialism/marketization (e.g., privatization, fee-paying recruitment, corporate/CME sponsorship, accreditation markets) and its intersection with neo-colonial dynamics (external credentialing dependence, knowledge hierarchies, migration-oriented training).
- ***Design:*** Qualitative (interviews, focus groups, ethnography, critical discourse analysis) or qualitatively analyzed mixed methods.
- ***Evaluation:*** Experiences, mechanisms, perceived impacts on curricula, assessment, access, quality, ethics.
***•Research type:*** Peer-reviewed Q1/Q2 journals (Scimago/JCR tiers), 2015-2025, English.

### Exclusions:

purely quantitative studies, opinion pieces/commentaries (used only for background), HIC-only settings without LMIC relevance, and non-Q1/Q2 venues.

### Information sources & search:

Databases searched (2015–2025) included MEDLINE/PubMed, Embase, Scopus, Web of Science, and ERIC; supplemented by targeted hand-searching of key journals, including Medical Education, Academic Medicine, BMC Medical Education, BMJ Global Health, Social Science & Medicine, Globalization and Health, and Medical Teacher. Search strings combined terms for medical education AND (commercial OR private OR market OR sponsor OR industry OR pharma OR fee OR for-profit OR accreditation OR agent OR recruitment) AND (LMIC/country names) AND (qualitative OR interview OR ethnography OR “discourse analysis”). Illustrative included records are cited in Table-I and below (representative studies include China MBBS recruitment, Pakistan CME providers, South Asian IMG discourses in the UK, and a private Mexican medical school case). Where applicable, articles available as ‘Epub ahead of print’ or early online release were included and dated according to journal indexing conventions.

**Table-I:** Summary of Included Qualitative Studies Exploring Commercialism and Neo-Colonial Influences in Medical Education.

Study (year)	Country / Setting	Journal (tier)	Design / Participants	Focus of Commercialism	Neo-colonial Link	Key Takeaways
Arfeen et al. (2024)^4^	India / Pakistan IMGs to UK	*BMJ Glob Health* (Q1)	Critical discourse analysis of archival texts	Credential dependence and migration markets	UK credentialing discourses position South Asian doctors in gratitude frames	Reinforces hierarchical knowledge flows; feeds back into LMIC curricula
Jiang et al. (2022)^8^	China (English-medium MBBS)	*BMC Med Educ* (Q2)	Qualitative survey + content analysis of international students	Fee-paying recruitment through agents and branding	Export of HIC curricula to LMIC students	Marketized recruitment creates quality-assurance pressures borne by students
Vakani et al. (2024)^9^	Pakistan (CME ecosystem)	*BMC Med Educ* (Q2)	Qualitative interviews with CME providers and faculty	Commercial sponsorship in CME and conflict of interest safeguards	HIC-modeled CME standards interact with local pharma landscape	Need for regulation and governance to protect educational integrity
Erana-Rojas et al. (2025)^11^	Mexico (private medical school)	*TQM Journal* (Q1 / Q2)	Qualitative case study (faculty and students)	Accreditation as market signal and branding tool	External standards orient curricula toward market evaluators	Accreditation perceived as necessary for competitiveness; risks curricular drift

*Legend:* This table summarizes the key characteristics, focus areas, and thematic contributions of the included qualitative studies.

*Note:* Journal quartile rankings (Q1/Q2) are based on Scopus 2025 metrics. Superscript numbers correspond to citations in the reference list.

### Study selection & data extraction:

Titles and abstracts were independently screened against predefined eligibility criteria, followed by full-text assessment of potentially relevant studies.

Data extraction included study setting, participant characteristics, methodological approach, identified commercialism and neo-colonial constructs, and key themes supported by participant quotations.

**Fig.1:**
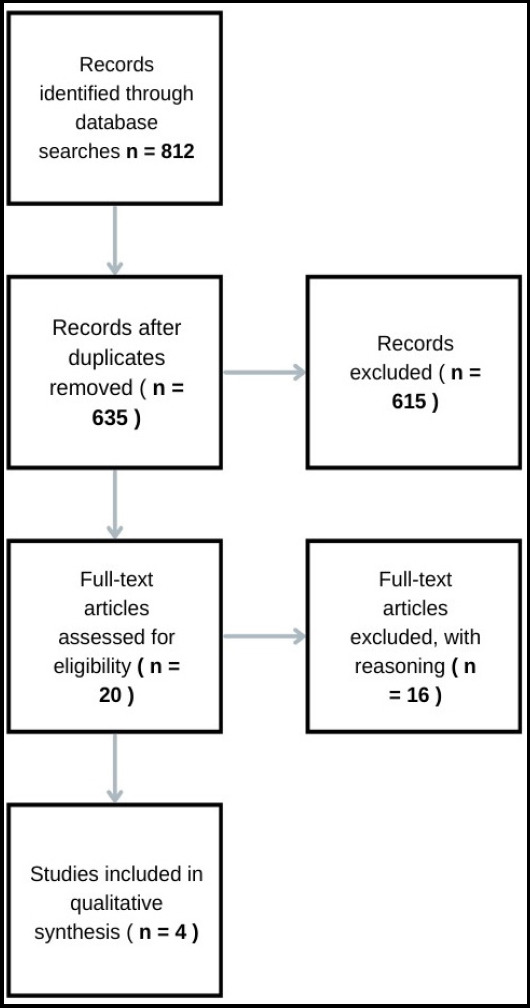
PRISMA 2020 flow diagram summarizing the identification, screening, eligibility, and inclusion of studies in this qualitative evidence synthesis.

Two reviewers independently screened and appraised all included studies using standardized criteria, and any discrepancies were resolved through discussion and consensus. Table-I summarizes the included qualitative studies, their settings, journal tier, design, and key insights related to commercialism and neo-colonial influences in LMIC medical education. A total of 812 records were identified through database searches. After removing duplicates, 635 records were screened based on titles and abstracts. Twenty full-text articles were assessed for eligibility, of which sixteen were excluded for not meeting the inclusion criteria. Four qualitative studies were included in the final synthesis (Fig.1).

### Methodological appraisal:

Used the CASP Qualitative Checklist to look at the methodological weaknesses in the studies (like the sampling, reflexivity, analysis transparency, and ethics). This assessment informed CERQual judgments but did not automatically exclude any studies.

### Synthesis & confidence assessment:

**Fig.2:**
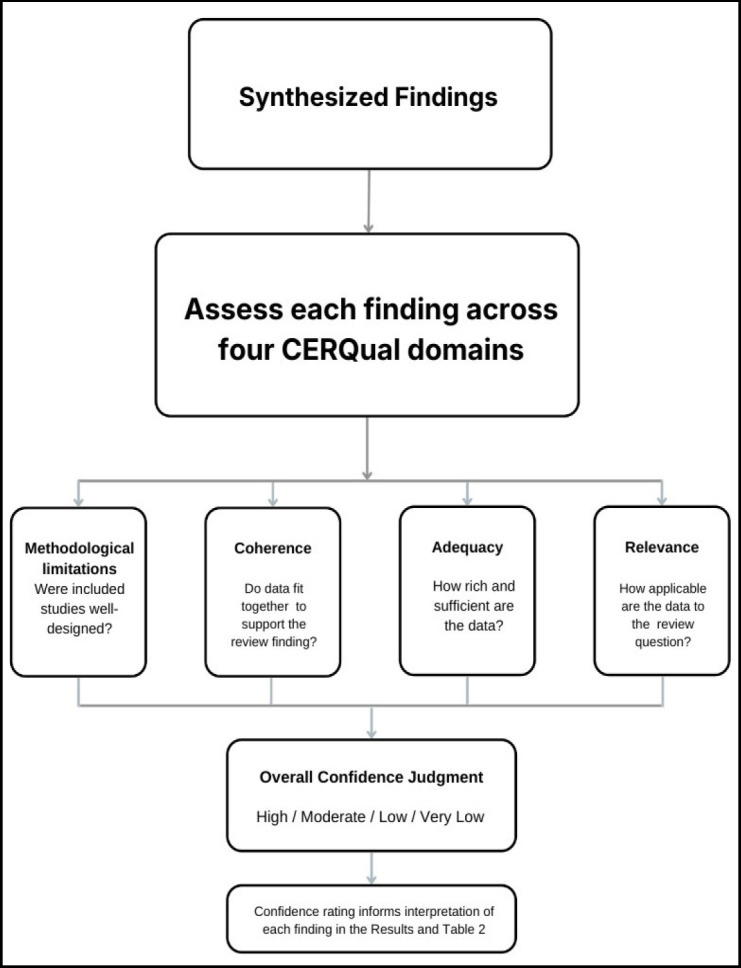
Overview of the GRADE-CERQual Assessment Process.

Data were coded line-by-line and grouped into descriptive and analytical themes. Then, we evaluated each synthesised finding using GRADE-CERQual, which assesses four key areas: methodological limitations, coherence, data adequacy, and relevance. The steps of this assessment are illustrated in Fig.2.

**Fig.3:**
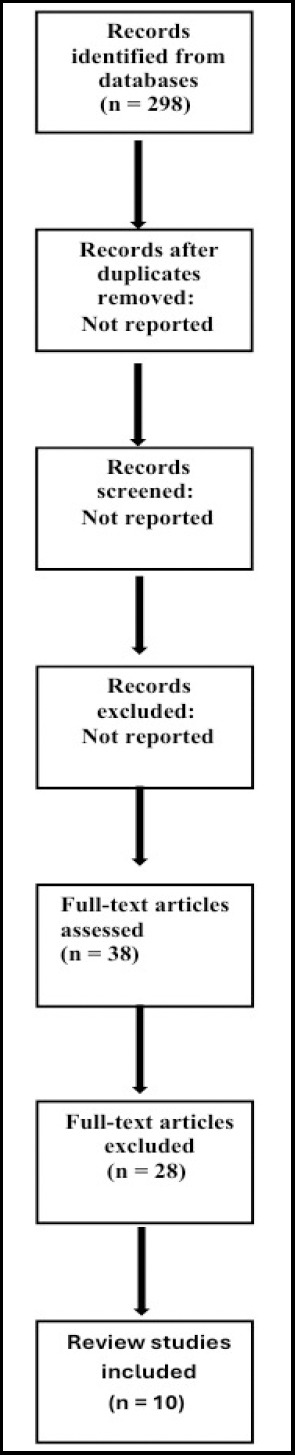
PRISMA

A Summary of Qualitative Findings (SoQF) was developed that includes confidence ratings and explanations for each finding. Table-II presents the GRADE-CERQual confidence ratings for each synthesized finding, outlining the level of confidence and the main concerns across domains. Eligible Q1/Q2 qualitative studies directly interrogating commercialization and neo-colonial mechanisms in LMIC medical education are limited. Our synthesis privileges depth and transparency over breadth and pairs this with explicit CERQual grading.^5^,^6^ The GRADE-CERQual framework was used to assess confidence in each synthesised finding by examining methodological limitations, coherence, adequacy of data, and relevance.^7^

**Table-II:** Summary of Qualitative Findings (SoQF) and GRADE-CERQual Confidence Assessments.

Finding	Summary of Evidence	CERQual Confidence Level	Explanation of Judgment
1. Credentialing dependence and migration markets	HIC credentialing systems (e.g., UK pathways) strongly influence learners and institutions in LMICs; training and assessment often focus on external credentials.	Moderate	Well-structured CDA with supporting studies; limited direct qualitative sources.
2. Market-driven cross-border recruitment	English-medium MBBS programmes recruit international fee-paying students via agents; marketing shapes perceptions of quality and recognition.	Moderate	Good qualitative evidence from China^8^; limited global coverage.
3. Commercial sponsorship in CME	CME systems reliant on pharma sponsorship introduce conflicts of interest; local providers call for governance reforms.	Moderate-High	Rich direct accounts from Pakistan^9^; high relevance to postgraduate education.
4. Accreditation as market signal	Accreditation seen as a quality marker but may prioritize indicators over community needs.	Low-Moderate	Based on single Mexican case study^11^; offers unique but limited contextual insight.

***Legend:*** Confidence ratings were determined using the GRADE-CERQual approach, based on methodological limitations, coherence, adequacy, and relevance.

***Note:*** CERQual = Confidence in the Evidence from Reviews of Qualitative Research. Superscript numbers correspond to the reference list.

Empirical qualitative studies from diverse LMIC contexts provide illustrative grounding for these analytical approaches. For example, qualitative research on English-medium medical programmes in China demonstrates how fee-paying international student recruitment, often mediated by commercial agents, reshapes curricula and quality assurance practices.^8^

Similarly, qualitative studies from Pakistan highlight tensions within continuing medical education (CME), where pharmaceutical sponsorship raises concerns regarding conflicts of interest and regulatory oversight.^9^

Synthesized Findings (with GRADE-CERQual):

*1. Credentialing dependence and migration markets play a significant role in shaping the priorities of medical education in low- and middle-income countries***.** HIC credentialing systems and discourses (e.g., UK pathways) strongly influence learners and institutions. Training and assessment practices often prioritize external credentials and anticipated high-income labour markets, reinforcing hierarchical knowledge flows.

**CERQual:** Moderate (well-structured CDA with supporting studies; limited direct qualitative data).

*2. Market-driven cross-border recruitment places students at financial and academic risk and exerts pressure on curricula and quality assurance mechanisms*. English-medium MBBS programmes recruit international students through fee-paying models often mediated by commercial agents, particularly affecting students from low- and middle-income countries financing their mobility. These pressures may shift institutional priorities toward revenue generation and student satisfaction rather than educational quality.

**CERQual:** Moderate (strong qualitative evidence in one context; limited geographic diversity).

*3. Commercial sponsorship in continuing medical education (CME) introduces conflicts of interest that require governance reforms*. In Pakistan, pharmaceutical industry involvement in CME raises concerns regarding influence on content, delivery, and professional independence. Participants emphasized the need for clear regulatory frameworks, standardized accreditation processes, and separation of education from marketing.

***CERQual:*** Moderate-to-High (rich, contextually grounded data; strong policy relevance).

*4. Accreditation functions as a market signal, guiding curricula towards easily interpretable indicators*. In a private Mexican medical school, accreditation is perceived as essential for competitiveness and institutional legitimacy. However, alignment with external indicators may lead to “teaching to the audit,” prioritizing compliance over responsiveness to local community needs.

**CERQual:** Low-to-Moderate (single-site case; contextually rich but limited generalizability).

## DISCUSSION

These findings should be interpreted within the constraints of a small but methodologically rigorous evidence base, consistent with qualitative evidence synthesis approaches that prioritize analytical depth over breadth.

Appendix: Summary of Included Studies (brief CASP appraisal).

• Arfeen 2024 (BMJ Global Health; CDA): Clear question; rigorous CDA with reflexive post-colonial lens; excellent coherence; relevance high to credentialing discourse; limitations: historical corpus, UK setting (implications inferred for LMICs).

• Jiang 2022 (BMC Med Educ): Appropriate qualitative approach; diverse international student sample; strong relevance to fee-paying cross-border programmes; limitation: single-country host context.

• Vakani 2024 (BMC Med Educ): Purposive sampling of CME stakeholders; rich, practice-proximal data; explicit policy recommendations; limitation: national focus.

Private Mexican school case (TQM Journal): Qualitative case with multi-stakeholder views; illuminates accreditation as market signal; limitation: single-site; journal outside edu category though ranked; transferability must be judged cautiously.

### How commercialism and neo-coloniality interact:

- ***Credential hierarchies and path-dependency:*** A critical discourse analysis of South Asian immigrants in the UK reveals how historical narratives of ‘opportunity’ and ‘gratitude’ continue to shape perceptions of legitimacy.^4^ These narratives subtly guide LMIC institutions toward emulating HIC standards and assessment systems, influencing curriculum priorities (e.g., exam-focused training for external licensing pathways) and framing outbound mobility as a marker of success. International credentials such as MRCP (UK), USMLE (USA), and PLAB remain highly sought after in LMICs, often functioning as gatekeepers to professional mobility and recognition. To operationalize regional accreditation strengthening, existing collaborative platforms may be leveraged and formalized. These include regional and international bodies such as the African Medical Schools Association (AMSA), South-East Asia Regional Association for Medical Education (SEARAME), and the Association for Medical Education in Europe (AMEE), which already support LMIC institutions through capacity-building, faculty development, and accreditation-related initiatives. Strengthening such platforms into formalized regional accreditation alliances could reduce dependence on Global North credentialing systems while maintaining contextually relevant quality benchmarks.
- ***Students’ behavior as customers in cross-border programmes:*** International students looking to study English-medium MBBS programmes often rely on recruitment agents and branding to secure their spots. These students become paying customers, and their expectations and anxieties about recognition can significantly impact the service orientation and reputation of the schools. While these factors may influence the overall experience, they do not necessarily lead to significant improvements in the quality of education. The qualitative evidence suggests that there might be some challenges in ensuring equal support, varying quality assurance, and complex recognition processes for graduates, particularly those from low-income countries.^8^
- ***Sponsorship capture and CME integrity:*** CME ecosystems that rely on industry funding are prone to certain conflicts of interest. These include topics that are chosen based on industry preferences, teaching methods that are influenced by brands, and a lack of clear distinction between education and marketing. Providers are pushing for policies that limit the involvement of businesses in educational settings. They are also advocating for independent needs assessments and professional accreditation to build trust.^9^ This aligns with global discussions about the role of businesses in health and offers a practical way to regulate these issues. Encouraging evidence-based medicine (EBM) practices and altruism among clinicians can mitigate commercial influences, ensuring that teaching and clinical decisions prioritize patient welfare over financial incentives.
- ***Accreditation as a market signal:*** In graduate markets, external labels are highly valued, so accreditation becomes both a way to ensure quality and a way to signal quality.^9^ In Mexico, qualitative accounts suggest that institutions adopt indicator regimes to secure market share and student mobility. Formalized quality assurance is in place, but the risk is that institutions focus so much on meeting audit requirements that they neglect local social accountability and equity goals which are essential for decolonizing agendas.^4^,^9^

Methodological scholarship on qualitative evidence synthesis emphasizes the importance of transparently linking analytical themes to confidence judgments and contextual interpretation, particularly when evidence bases are small or heterogeneous.^10^ Empirical qualitative case studies of private medical schools further illustrate how accreditation operates as a competitive market signal, shaping institutional priorities, faculty behavior, and curricular alignment in ways that may conflict with local social accountability goals.^11^

Some countries, including South Africa, India, and Pakistan, have initiated reforms to decolonize medical education by prioritizing community-based curricula and national accreditation standards aligned with local health priorities. Similar concerns regarding accreditation, quality assurance, and regulatory capacity have also been reported within Pakistani medical education systems.^12^

### Equity and social accountability implications:

Commercial interests can sometimes make it harder to provide education that’s responsive to the needs of the community. This can lead to unfair differences in access to education, like higher fees, coaching costs, and different opportunities to earn recognized credentials. Social accountability is a strong part of our curricula and governance, but efforts must be made so that it’s not influenced by things like branding and recruitment growth, which could create some strange incentives.

### Evidence gaps:

Although commercial pressures such as entrance-examination coaching industries are widely reported, there is a notable absence of rigorous Q1/Q2 qualitative studies examining these phenomena within medical education systems. We identified few Q1/Q2 empirical qualitative studies directly interrogating commercialization and neo-colonial mechanisms within LMIC medical education.

Notably, few multi-country ethnographies were identified. There’s also not much qualitative work on the coaching industry’s impact, and studies on the opportunity costs of accreditation in resource-limited settings are scarce. In future research, it’s important to focus on comparative designs that involve multiple sites. We should also make sure to explicitly theorize about the intersections of colonialism and markets.

### Methodological Reflections and Limitations:

- Although the review followed a structured qualitative evidence synthesis approach, the limited number of eligible studies constrained the breadth of included contexts. However, dual independent screening, transparent analytic procedures, and formal CERQual assessment were maintained to enhance rigor.
- Publication bias: Q1/Q2 restriction improves quality but might not be picking up all the qualitative work that’s published in smaller outlets, especially in specific regions.
- Scope: Focused on studies that directly linked commercialization to neo-colonial dynamics. Also used other related fields (like broader social accountability and virtual learning markets) as context.
- A key limitation is the inclusion of only four studies meeting strict Q1/Q2 qualitative criteria. Importantly, this limits not only generalizability but also geographic representativeness. The findings should therefore be interpreted as exploratory and hypothesis-generating, intended to inform future multi-country qualitative research rather than to make definitive claims about all LMIC contexts. While this enhances methodological rigor, it necessarily constrains the breadth of contexts represented in the synthesis.

### Implications for Policy and Practice:

1.Commercial involvement in medical education should be appropriately regulated, especially with CME. Have independent assessments to check if these activities are fair, make sure that sponsors don’t control the content, and be transparent about who’s getting paid. Also, make sure that educational providers are properly accredited.
2.Reduce how much we rely on credentials by forming strong alliances with regional assessment and accreditation bodies. This way, it can be ensured that quality is not solely determined by high-income countries.
3.Make sure international student markets are run ethically. We need to be transparent about how students can get recognized and how we’re ensuring quality. We should also limit the incentives that agents must push students to certain programmes. And finally, we should put in place student protection policies for cross-border programmes.
4.Shift our focus to social accountability metrics like community-responsive outcomes and equitable access. These measures are more meaningful than just market-oriented indicators.

### What this meta-synthesis adds:

- Four main ways commercialism and neo-colonialism mix in medical education in low- and middle-income countries (LMICs) These are:
- ***1.Credential dependence:*** Doctors need to get degrees from certain institutions to practice.
- ***2.Cross-border recruitment markets:*** Medical students are increasingly recruited across borders through fee-paying models, particularly within South Asia.
- 3.Cross-border student recruitment has intensified in South Asia, with increasing reliance on fee-paying models that expose students and families to significant financial risks. In India, the rapid growth of private “coaching factories” and examination-related corruption further illustrates how commercial pressures can distort access to medical education.
- ***4.Sponsorship capture:*** Doctors who receive funding from foreign companies often must follow their interests.
- ***5.Accreditation signaling:*** Doctors who have degrees from accredited institutions are often perceived as more competent, trustworthy, and professionally credible, which enhances their career opportunities and patient confidence.

### Policy Advice:

Offers practical policy changes (CME COI rules; student protection in recruitment; rebalanced accreditation metrics; regional credential alliances).

## CONCLUSION

This qualitative evidence synthesis highlights how commercial and neo-colonial forces intersect to shape medical education in low- and middle-income countries. Credential dependency, market-driven recruitment, commercial sponsorship, and accreditation as market signals reflect enduring structural imbalances. Strengthening regional accreditation, regulating industry involvement, and prioritizing social accountability can help decolonize and rebalance medical education systems. Future research should focus on multi-country qualitative comparisons and explore sustainable, context-responsive governance models.

## Data availability:

All data relevant to the study are included in the article or uploaded as supplementary information. No new datasets were generated.

### Author’s contribution:

All authors made a significant contribution to the work reported, whether that is in the conception, study design, execution, acquisition of data, analysis and interpretation, or in all these areas; took part in drafting, revising or critically reviewing the article; gave final approval of the version to be published; have agreed on the journal to which the article has been submitted; and agree to be accountable for all aspects of the work.
